# Ensuring Nutritious Food Under Elevated CO_2_ Conditions: A Case for Improved C_4_ Crops

**DOI:** 10.3389/fpls.2020.01267

**Published:** 2020-08-18

**Authors:** Timothy O. Jobe, Parisa Rahimzadeh Karvansara, Ivan Zenzen, Stanislav Kopriva

**Affiliations:** Institute for Plant Sciences, Cluster of Excellence on Plant Sciences (CEPLAS), University of Cologne, Cologne, Germany

**Keywords:** sulfur, nitrogen, phosphorus, C_4_ photosynthesis, maize, *Flaveria*, hidden hunger

## Abstract

Global climate change is a challenge for efforts to ensure food security for future generations. It will affect crop yields through changes in temperature and precipitation, as well as the nutritional quality of crops. Increased atmospheric CO_2_ leads to a penalty in the content of proteins and micronutrients in most staple crops, with the possible exception of C_4_ crops. It is essential to understand the control of nutrient homeostasis to mitigate this penalty. However, despite the importance of mineral nutrition for plant performance, comparably less is known about the regulation of nutrient uptake and homeostasis in C_4_ plants than in C_3_ plants and mineral nutrition has not been a strong focus of the C_4_ research. Here we review what is known about C_4_ specific features of nitrogen and sulfur assimilation as well as of homeostasis of other essential elements. We identify the major knowledge gaps and urgent questions for future research. We argue that adaptations in mineral nutrition were an integral part of the evolution of C_4_ photosynthesis and should be considered in the attempts to engineer C_4_ photosynthetic mechanisms into C_3_ crops.

## Introduction

As global population continues to increase, crop yields must increase proportionally to meet the future demand for food. However, the quantity of food is not the only threat to food security, but also the nutritional quality of the food produced (Myers et al., 2017). Indeed, micronutrient deficiencies are estimated to affect over 2 billion people worldwide (Amoroso, 2016). Thus, micronutrient deficiencies impinge on agricultural production, food security, and human health. Global climate change is another factor negatively influencing crop nutritional quality. Many crops grown under the predicted elevated atmospheric CO_2_ concentration show an increase in yield, but a decrease in micronutrients (zinc, iron) and proteins (as nitrogen) (Loladze, 2014; Zhu et al., 2018; Ujiie et al., 2019). This decrease is partly due to an increased synthesis of carbohydrates at the expense of proteins, often referred to as the carbon dilution effect. However, it is also caused by the immobilization of nitrogen in vegetative tissues and soil (Luo et al., 2004) and by direct reduction in nitrate assimilation by elevated CO_2_ (Bloom et al., 2010). Interestingly, at least for rice, the decreased protein and nitrogen content observed is not completely due to a general carbon dilution, but due to differential responses of the superior grains (derived from early flowers) and the inferior grains (derived from late flowers) to elevated CO_2_ (Zhang et al., 2013). Nitrogen content decreases in superior grains, but it does not change in inferior grains. However, inferior grains are frequently lost during harvest, which further decreases the total grain protein yield (Zhang et al., 2013). Decreased protein content in crops means sulfur will also be less available for human nutrition as plant proteins are the primary source of the essential sulfur-containing amino acid methionine (Parcell, 2002). Indeed, independent FACE (free‐air CO_2_ enrichment) experiments in wheat showed a 7% decrease in total grain sulfur and an 8% decrease in methionine and cysteine content (Hogy et al., 2009; Fernando et al., 2012). This nutrient penalty has been observed for multiple crops, with one notable exception—C_4_ crops (Myers et al., 2014). Presumably, because C_4_ crops profit much less from elevated CO_2_ as carbon uptake in C_4_ plants is saturated at ambient CO_2_ levels (Von Caemmerer and Furbank, 2003), no carbon dilution effect occurs, and the elevated CO_2_ does not affect protein and micronutrient levels. Thus, C_4_ crops have great potential to deliver sufficient nutrients for human food and health. However, more effort is needed to understand the control of nutrient fluxes and homeostasis in C_4_ plants to ensure that this will also be true in the coming decades.

Compared to C_3_ crops, such as rice, wheat, or oil-seed rape, less is known about specific alterations in mineral nutrition of C_4_ plants, despite substantial differences in the organization of nitrate and sulfate assimilation (Jobe et al., 2019). Therefore, in this review, we summarize what is known about C_4_ specific features of nitrogen and sulfur metabolism as well as of homeostasis of other essential elements. To identify the major knowledge gaps and urgent questions for future research, we relate the current knowledge of plant mineral nutrition in C_3_ vs. C_4_ plants with future needs for human nutrition and health and with the predicted changes in atmospheric CO_2_ levels. Finally, we discuss future directions and approaches to prevent additional declines in the nutritional quality of crops, mainly engineering C_4_ photosynthetic mechanisms into C_3_ crops.

## C_4_ Photosynthesis and Plant Nutrition

**Figure 1:**
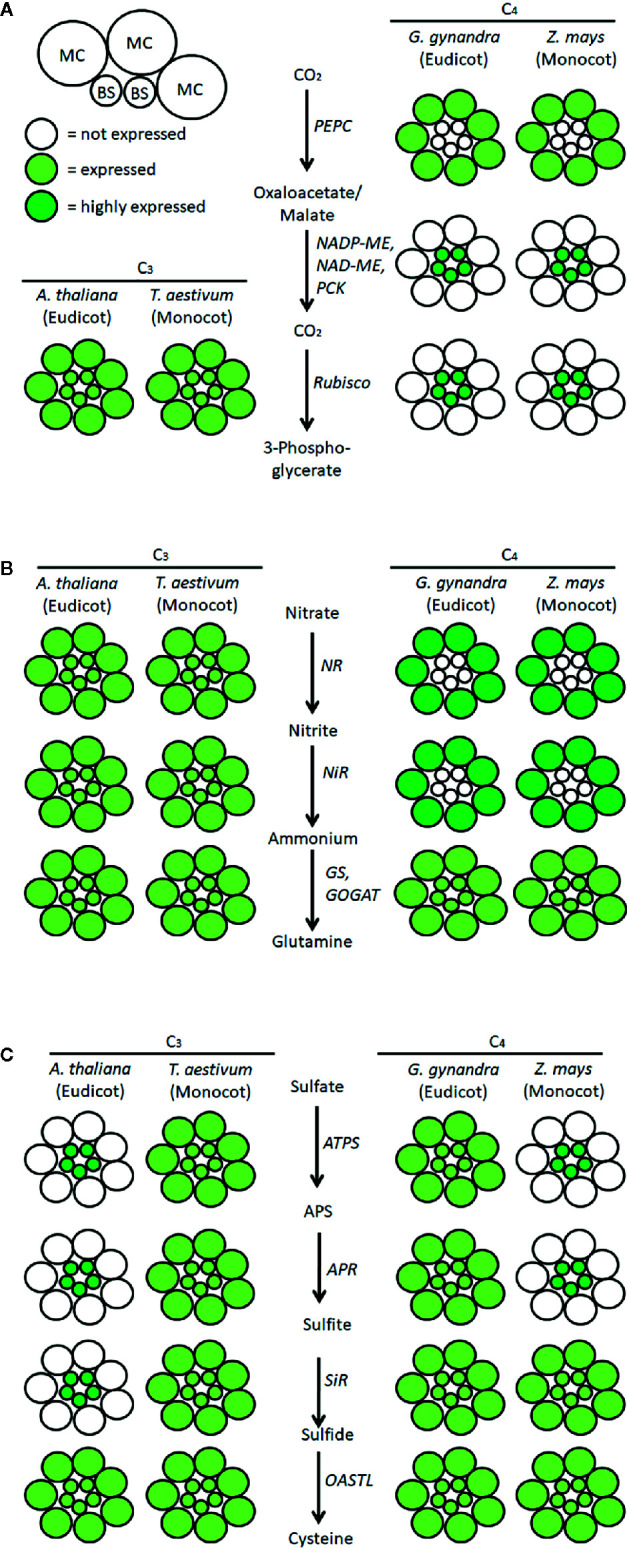
Scheme of localization of the pathways of carbon, nitrate, and sulfate assimilation in exemplary C_3_ and C_4_ plants. Schematic localization of key enzymes of **(A)** CO_2_, **(B)** nitrate, and **(C)** sulfate assimilation in mesophyll cells (MC) and bundle sheath cells (BS) of representative species for C_3_ and C_4_ monocots and dicots was compiled from the literature described in the manuscript. MC, mesophyll cells; BS, bundle sheath cells; PEPC, phosphoenolpyruvate carboxylase; ME, malic enzyme; PCK, phosphoenolpyruvate carboxykinase; NR, nitrate reductase; NiR, nitrite reductase, GS, glutamine synthetase; GOGAT, glutamate synthase; ATPS, ATP sulfurylase; APS, adenosine 5´-phosphosulfate; APR, APS reductase; SiR, sulfite reductase; OASTL, O-acetylserine (thiol)lyase.

Rubisco, the enzyme responsible for assimilating CO_2_ into reduced carbon compounds, is an inefficient catalyst under the current atmospheric conditions (Parry et al., 2013; Pottier et al., 2018; Ashida et al., 2019). This inefficiency arises because the carboxylase function of Rubisco can be competitively inhibited by atmospheric oxygen. Thus, many photosynthetic organisms have evolved CO_2_ concentrating mechanisms to boost the efficiency of Rubisco by increasing the concentration of CO_2_ at the site of carboxylation. Plants using the C_4_ photosynthetic pathway accomplish this by dividing the photosynthetic process into two specialized cell types (**Figure 1**). Within mesophyll cells (MC), the initial CO_2_ fixation step occurs *via* carboxylation of phosphoenolpyruvate using the enzyme phosphoenolpyruvate carboxylase (PEPC) (Hatch and Slack, 1966; Slack and Hatch, 1967). This is an essential step because PEPC is not inhibited by atmospheric oxygen. The product of this reaction is a four-carbon organic acid that then moves into the bundle sheath (BS) cells, where it is decarboxylated, releasing CO_2_ for Rubisco. Because of the low oxygen environment in the BS cells, Rubisco can operate near its maximal efficiency. This pathway has evolved independently in angiosperms at least 66 times, representing three families of monocots and 16 families of dicots (Von Caemmerer and Furbank, 2003; Sage et al., 2012).

While these independent C_4_ lineages share many characteristics, there are also significant differences in the C_4_-acid decarboxylase enzymes. These differences allow us to classify C_4_ plants into three biochemical subgroups. Plants that use NAD malic enzyme (NAD-ME) decarboxylate C_4_ acids in the BS mitochondria, while plants using NADP malic enzyme (NADP-ME) decarboxylate C_4_ acids in the BS chloroplasts. The third C_4_ subtype uses phosphoenolpyruvate carboxykinase (PCK) to decarboxylate C_4_ acids primarily in the cytosol of the BS cells. While all of these result in enhanced Rubisco efficiency, these biochemical subtleties reflect differences in the genetic prerequisites for C_4_ evolution as well as differences in the selective pressures that favored one subtype over another (Pinto et al., 2016; Sonawane et al., 2017). For example, within C_4_ grasses, NADP-ME plants increase in abundance geographically with increasing rainfall, while the number of NAD-ME grasses decreases in these conditions (Taub, 2000; Cabido et al., 2008). Thus, since the discovery of the C_4_ photosynthetic pathway, many studies have focused on identifying differences between C_3_ and C_4_ plants and between different C_4_ subtypes to unravel the genetics and evolution of C_4_ photosynthesis. Interestingly, nitrogen appears to be an essential component in many of these studies.

### Nitrogen

Early research in Poaceae noted that C_4_ grasses contained less total nitrogen in their leaves and produced more dry matter per unit of nitrogen fertilizer applied than C_3_ grasses. These observations quickly led to the hypothesis that C_4_ species utilize nitrogen more efficiently than C_3_ species (Brown, 1978). The obvious explanation was the lower investment of nitrogen in Rubisco in C_4_ plants (Sage et al., 1987). While this hypothesis is broadly accepted, recent studies suggest minor refinements are justified. For example, Ghannoum et al. (2005) evaluated combinations of various NAD-ME and NADP-ME grass species under high and low nitrogen treatments. They found that while the net CO_2_ assimilation rates were similar between these two C_4_ subtypes, NAD-ME plants contained more leaf nitrogen than NADP-ME plants with comparable CO_2_ assimilation rates. By measuring the total nitrogen in the MC and BS cells, Ghannoum et al. (2005) also showed that in the NAD-ME species, BS cells contained approximately 60% of the nitrogen and chlorophyll. In comparison, only 35% of the total nitrogen and chlorophyll were found in the BS of NADP-ME plants. Analysis of N partitioning suggested that NAD-ME plants invest more nitrogen into the production of Rubisco and other soluble proteins than NADP-ME plants. This seemed to be compensated by significantly greater k_cat_ values of Rubisco in NADP-ME than in NAD-ME species (Ghannoum et al., 2005). Furthermore, a systematic evaluation of several lineages of C_4_ grasses encompassing all three biochemical C_4_ subtypes found that the nitrogen use efficiency of C_4_ grasses is highly correlated with the biochemical subtype with NADP-ME and PCK grasses having higher nitrogen use efficiency than NAD-ME counterparts (Pinto et al., 2016). Thus, while C_4_ plants have a higher photosynthetic nitrogen use efficiency (PNUE) than C_3_ plants, different biochemical C_4_ subtypes vary in their PNUE.

Nitrogen assimilation in C_4_ plants differs from C_3_ plants not only in PNUE but also in the intercellular compartmentalization of nitrogen assimilation enzymes (**Figure 1**; Kopriva, 2011; Jobe et al., 2019). Already at the onset of C_4_ photosynthesis research, it was shown that the activity of nitrate reductase, the key enzyme of nitrate assimilation, is localized mainly in the MC of maize, *Sorghum sudanense*, and *Gomphrena globosa* (Mellor and Tregunna, 1971). Further studies including all three C_4_ metabolic subtypes revealed that nitrate reductase was coordinately localized with nitrite reductase, glutamine synthetase, and glutamate synthase, in MC of maize, *Sorghum bicolor*, *Digitaria sanguinalis*, and *Panicum miliaceum*, while in *Panicum maximum*, nitrite reductase was present both in MC and BS (Rathnam and Edwards, 1976). Other studies confirmed the predominant localization of nitrate reductase and nitrite reductase activities in MC, but glutamine synthetase and glutamate synthase were mostly found in both MC and BS (Harel et al., 1977; Moore and Black, 1979). Immunogold labeling confirmed the exclusive localization of maize nitrate reductase in the cytosol of MC (Vaughn and Campbell, 1988) and glutamine synthetase and glutamate synthase in both cell types (Becker et al., 2000). However, whether the spatial distribution of nitrate assimilation in C_4_ plants contributes to their PNUE is unknown.

These observations prompted researchers to evaluate the potential role of nitrogen use efficiency and nitrate assimilation as drivers for the evolution of C_4_ photosynthesis. Classical schematic models of C_4_ evolution suggest that ancestral C_3_ plants progressed through a series of discrete stages on the path to C_4_ photosynthesis (Edwards et al., 2001; Heckmann et al., 2013; Schluter and Weber, 2016). The first stage is an increase in the BS : MC ratio driven by CO_2_ limitation or other environmental factors and a reallocation of glycine decarboxylase (GDC) expression from the MC to the BS. Next is the establishment of C_2_ photosynthesis (Mallmann et al., 2014). Next, there is an upregulation of the photorespiratory genes in both the BS and MC, a decrease in Rubisco expression in the MC, and an upregulation of PEPC in the MC (Schluter and Weber, 2016). In the final evolutionary stages, the expression of Rubisco and the photorespiratory genes become confined to the BS. Recent advances in constraint-based modeling have enabled researchers to examine the selective pressures that lead to C_4_ photosynthesis *in silico* (Blatke and Brautigam, 2019). These analyses suggested that while light and light distribution were the main drivers governing choice of decarboxylation enzymes, they also predicted that nitrogen limitation might have contributed to C_4_ evolution under high levels of photorespiration (Blatke and Brautigam, 2019).

What advantages do these evolutionary adaptations give to C_4_ plants over C_3_ plants as atmospheric CO_2_ increases? Nitrate assimilation was shown to be inhibited by elevated CO_2_ in a number of C_3_ species but not C_4_ plants (Hocking and Meyer, 1991; Bloom et al., 2012). Elevated CO_2_ increased PNUE of wheat but not maize, particularly at lower nitrate input, due to enhancing growth, however, at the expense of N accumulation in leaves (Hocking and Meyer, 1991). Nitrate reductase activity was inhibited by the elevated CO_2_ in wheat and not in maize (Hocking and Meyer, 1991). Nitrate assimilation can be quantified *in vivo* by an assimilatory quotient, the ratio of net CO_2_ consumed over net O_2_ evolved (Bloom et al., 2012). Plants assimilating nitrate increase net O_2_ evolution while CO_2_ consumption is constant, therefore, the assimilatory quotient is low in plants reducing nitrate (Bloom et al., 1989). The quotient is usually determined in comparison with ammonium nutrition after the addition of nitrate as a ΔAQ. In a number of C_3_ plants ΔAQ was high at low CO_2_ concentrations, but rapidly diminished with increasing CO_2_ in accordance with inhibition of nitrate reductase by elevated CO_2_ (Bloom et al., 2012). In contrast, in three C_4_ species analyzed, the ΔAQ was lower at low CO_2_ levels but remained constant with increasing CO_2_. Interestingly, in C_3_–C_4_ intermediate plants the response of ΔAQ to CO_2_ was intermediate between C_3_ and C_4_. Accordingly, FACE experiments have consistently shown that increasing CO_2_ negatively impacts nitrogen levels in C_3_ plants. This is true for leaves, where often, but not always, Rubisco content diminishes (Bowes, 1991) and for seeds and grains. A recent meta-analysis showed that the average differential effect of increased CO_2_ on C_3_ plants is—4% (Ebi and Loladze, 2019). In a comparison between several C_3_ crops, Myers et al. (2014) found no significant changes in nitrogen content in maize grown under elevated CO_2_. While it remains unclear if nitrogen limitation contributed to C_4_ evolution, the rising CO_2_ levels do not pose a threat for a reduction in nitrogen in C_4_ plants as they are already saturated at current CO_2_ levels (Von Caemmerer and Furbank, 2003). Although the lower abundance of Rubisco and the identity of the decarboxylation enzyme were shown to impact nitrogen use efficiency, less is known regarding the significance of confining nitrate reduction to the MC. However, it highlights the extensive metabolic rewiring that accompanies C_4_ evolution and suggests that multiple mechanisms contribute to enhanced nitrogen use efficiency in C_4_ plants. Taken together, both recent and historical studies show that C_4_ plants require less total nitrogen, have higher nitrogen use efficiency, and maintain nitrogen levels under elevated CO_2_ conditions.

### Sulfur

Sulfur is an essential macronutrient for all living organisms, with organic S-compounds representing an important class of metabolites in plant physiology. Sulfate assimilation by plants and microorganisms constitute the entry point of this element into organic molecules in the global sulfur cycle and also in human nutrition. Sulfate is the primary source of S available in nature, and specific H+/sulfate co-transporters from the SULTR family mediate sulfate uptake and mobilization within the plant (reviewed in Takahashi et al., 2011a; Gigolashvili and Kopriva, 2014). Once inside the plant cell, sulfate is initially activated by ATP sulfurylase (ATPS), producing adenosine 5′-phosphosulfate (APS). In primary S-metabolism, APS undergoes two subsequent reduction reactions catalyzed by APS reductase (APR) to generate sulfite and sulfite reductase (SiR) to produce sulfide. Finally, in a two-step process, serine acetyltransferase catalyzes the transfer of an acetyl moiety from acetyl Coenzyme A to serine resulting in *O*-acetyl-L-serine (OAS). OAS is then used as a substrate for *O*-acetylserine(thiol)lyase (OASTL), which replaces the acetyl group of OAS with sulfide to produce cysteine, the first organic form of sulfur (reviewed in Takahashi et al., 2011b). Cys is the source of reduced S for other metabolites, such as methionine or the tripeptide glutathione (GSH), an essential part of plant redox homeostasis and stress defense (Noctor et al., 2012).

Like nitrate assimilation, sulfate assimilation is differentially localized in MC and BS of C_4_ plants. In a number of C_4_ species spanning all three C_4_ subtypes, most of the total leaf ATPS activity is confined to BS chloroplasts (Gerwick et al., 1980; Passera and Ghisi, 1982). Similar to nitrate assimilation, not all enzymes of the pathway are coordinately expressed. While APR was also found almost exclusively in BS of maize (Schmutz and Brunold, 1984; Burgener et al., 1998), the activities of SiR and OASTL were detected at comparable levels in MC and BS (Passera and Ghisi, 1982; Schmutz and Brunold, 1985). Reduced sulfur needed in MC is transported from maize BS in the form of cysteine (Burgener et al., 1998). Interestingly, GSH synthesis and homeostasis are also differently organized in MC and BS. In maize, GSH synthetase activity is higher in MC than in BS, in line with the export of Cys from BS (Burgener et al., 1998). This results in a higher accumulation of GSH in MC, possibly connected to higher H_2_O_2_ levels in MC than in BS (Doulis et al., 1997). Given the importance of GSH for maintaining cellular redox potential, it is surprising that glutathione reductase, the key element of the glutathione redox cycle, was also found exclusively in MC of maize (Doulis et al., 1997; Pastori et al., 2000). However, not all C_4_ plants follow the same pattern. In the C_4_ species of the dicot genus *Flaveria*, APR and ATPS are expressed in both MC and BS (Koprivova et al., 2001). Since the C_4_ species analyzed previously were all monocots, BS-exclusive localization of sulfate assimilation could be a trait of C_4_ monocots but not C_4_ eudicots (**Figure 1**; Koprivova et al., 2001; Kopriva and Koprivova, 2005). Indeed, numerous RNA-seq analyses of MC and BS transcripts showed BS localization of transcripts for ATPS and APR in different C_4_ monocots (maize, sorghum, *Setaria viridis*) but a similar transcript abundance in MC and BS of the eudicot C_4_ species *Gynandropsis gynandra* (Aubry et al., 2014a; John et al., 2014; Doring et al., 2016; Denton et al., 2017). Thus, the localization of sulfate assimilation in BS cannot be a general C_4_ trait. This conclusion was unexpectedly confirmed by experiments with the C_3_ model plant, *Arabidopsis thaliana*. In an analysis aimed at discerning the function of the BS cell layer in C_3_ plants using a translatome approach, Aubry et al. (2014b) found an enrichment of transcripts for sulfate assimilation genes in the BS. Transcripts of ATPS, APR, SiR, as well as other components of Cys synthesis, in addition to sulfate transporters and genes for synthesizing the sulfur-rich secondary compounds glucosinolates were all overrepresented in RNA from BS compared to the whole leaf (Aubry et al., 2014b). Three obvious questions arise from this study. First, what is the ancestral localization of the sulfate assimilation pathway? Secondly, what is the metabolic significance of the various relocations? Finally, in the C_4_ lineages with relocated sulfate assimilation enzymes, was the relocation of sulfate assimilation a prerequisite for C_4_ evolution or a consequence of C_4_ evolution? These remain key open questions in plant sulfur research.

An analysis of sulfate assimilation in the eudicot genus *Flaveria* revealed another intriguing result. A gradient in the accumulation of leaf Cys and GSH was observed with higher concentrations in the leaves of C_4_ species than in C_3_ and C_3_-C_4_ intermediate species (Koprivova et al., 2001; Gerlich et al., 2018). This gradient is sustained through a similar gradient in sulfate uptake, reduction rate, transcript levels, and activity of APR (Koprivova et al., 2001; Weckopp and Kopriva, 2014; Gerlich et al., 2018). Interestingly, expression analyses suggested that sulfate reduction and GSH synthesis are preferentially localized in the roots of C_4_ *Flaveria* species. Interspecies grafts of C_3_ *F. robusta* and C_4_ *F. bidentis* were created to test this hypothesis. The results of this experiment showed that the high GSH accumulation in C_4_ leaves is indeed controlled by the roots (Gerlich et al., 2018). While it is plausible that the importance of roots for Cys and GSH synthesis in C_4_ *Flaveria* is connected to serine synthesis, which is preferentially synthesized in the roots of C_4_ plants through the phosphorylated pathway (Gerlich et al., 2018), this hypothesis should be tested in more C_4_ species.

Sulfur is much less abundant in the plant body than nitrogen making it unlikely to be the driving force behind the metabolic adaptations leading to the evolution of C_4_ photosynthesis. However, it is possible that the gradient of higher sulfate assimilation flux with increasing C_4_ photosynthesis in *Flaveria* is a result of the adaptation to dry and warm habitats typical for C_4_ plants. Thus, the higher GSH contents in C_4_ *Flaveria* might be a mechanism to cope with increased oxidative stress caused by such environmental conditions. This is consistent with the critical role of GSH in chilling tolerance in maize (Kocsy et al., 2001). However, the importance of the BS-localization of sulfate assimilation in C_4_ monocots and possibly in the roots of C_4_ dicots is still elusive.

### Phosphorus

In addition to carbon, nitrogen, and sulfur, phosphorus is a macronutrient crucial for plant growth and development. As an essential player in cellular energy conversion, an enzymatic substrate, as well as a regulatory factor of enzyme activity, phosphate plays many crucial roles in cellular biochemistry. Moreover, phosphate is responsible for the acidic nature of nucleic acids and is a vital constituent of phospholipid membranes. Plants employ several morphological and physiological adaptations to mitigate phosphorus deficiency, including interconnections with the rhizosphere and soil microbes and diverse molecular mechanisms (Lopez-Arredondo et al., 2014). Phosphate is taken up by various phosphate transporters as an inorganic anion. However, unlike nitrate and sulfate, phosphate is not reduced and remains in its oxidized state as either a free anion or is incorporated into organic compounds *via* phosphate esters. Disruptions in phosphate homeostasis have intensive footprints on plants. Thus, shoot phosphate concentrations are tightly regulated by systemic control of phosphate uptake and allocation (Bari et al., 2006; Ham et al., 2018; Kopriva and Chu, 2018). Control of phosphate homeostasis is coordinated with the regulation of other nutrients, particularly nitrate and sulfate (Rouached et al., 2011; Hu et al., 2019; Medici et al., 2019).

Phosphate has a vital role in photosynthesis. The metabolic energy of the cell and the energy generated during the light reactions of photosynthesis are stored in phosphate esters and energy-rich pyrophosphate bonds. Inorganic phosphate in the chloroplast regulates the partitioning of photosynthates between starch synthesis and export to the cytosol (Heldt et al., 1977). Moreover, phosphate is indispensable for the function of the triose-phosphate/phosphate translocator (TPT), an antiporter in the inner membrane of the chloroplast (Lee et al., 2017). The TPT exchanges phosphate from the cytosol with triose-phosphates synthesized in the Calvin cycle (Fliege et al., 1978). In C_4_ plants, the TPT is even more highly abundant in envelopes of MC chloroplasts as the flux through this transporter is higher in C_4_ plants than in C_3_ plants (Brautigam et al., 2008). In addition, C_4_ plants possess another abundant phosphate driven transporter, the phosphoenolpyruvate phosphate translocator (PPT), which is essential for the transport of PEP from the chloroplast in MC (Brautigam et al., 2008; Majeran et al., 2008). Also, the activities of the critical enzymes involved in C_4_ carbon assimilation, such as PEPC, PCK, and pyruvate phosphate dikinase, are modulated by reversible phosphorylation (Ashton and Hatch, 1983; Jiao and Chollet, 1991; Chao et al., 2014).

Although phosphate demand to facilitate transport processes in C_4_ plants is high, C_4_ specific features of phosphate homeostasis or possible differences in (photosynthetic) phosphate use efficiency (PUE) have not been described. Phosphate deficiency was shown to decrease Rubisco activity in sunflower, but Rubisco activity was not affected by phosphate deficiency in maize (Jacob and Lawlor, 1992). Similarly, C_4_ grasses produced higher forage yields on phosphate-limited soil than C_3_ grasses (Morris et al., 1982). Accordingly, in a comparative survey of photosynthetic and growth responses to phosphate deficiency in 12 species with diverse photosynthetic characteristics, C_3_ species showed more substantial growth retardation in comparison to C_4_ species (Halsted and Lynch, 1996). However, no photosynthesis type-dependent changes in photosynthetic PUE could be determined. Although the CO_2_ exchange rate was decreased less by phosphate deficiency in C_4_ plants than in C_3_ ones, due to higher foliar phosphate concentration, the photosynthetic PUE remained unchanged (Halsted and Lynch, 1996). Interestingly, the monocot species were less sensitive to low phosphate stress than dicots irrespective of photosynthesis type, due to a lower phosphate content in the leaf and better maintenance of growth (Halsted and Lynch, 1996). In an independent study focusing on monocots, the response of CO_2_ assimilation rates to leaf phosphate concentration was saturated in C_4_ species but not in their C_3_ relatives (Ghannoum et al., 2008). It seems, therefore, that although C_4_ plants require higher amounts of phosphate than C_3_ plants, their CO_2_ assimilation is less sensitive to phosphate limitation.

## How Does Elevated CO_2_ Affect Micronutrients in C_3_ and C_4_ Plants?

The World Health Organization (WHO) defines malnutrition as deficiencies, excesses, or imbalances in a person´s energy intake and/or nutrient intake (https://www.who.int/news-room/fact-sheets/detail/malnutrition) and recognizes three broad groups of malnutrition conditions - undernutrition, micronutrient-related malnutrition, and overnutrition and noncommunicable diseases. Over the past 60–70 years, plant biologists and plant breeders have focused their attention on alleviating undernutrition by dramatically increasing crop yields by improving plant genetics and intensifying agricultural production systems. However, by focusing on yield, changes in the nutritional value of our food have been largely neglected, especially regarding micronutrient content. Thus, micronutrient levels in plants have decreased for two main reasons. First, intensive agricultural practices have depleted micronutrients from the soil, and second, rising atmospheric carbon dioxide negatively affects the nutrient profiles of C_3_ crop plants (Loladze, 2014).

Micronutrient-related malnutrition, sometimes called hidden hunger, is caused by poorly diversified diets that meet the caloric but not the nutritional needs of an individual and is primarily associated with micronutrient deficiency (Myers et al., 2017). In addition to the well documented adverse effects of increasing atmospheric CO_2_ on macronutrients in C_3_ crops (see above), there is evidence that the effects are equally adverse, or in some cases, much worse for micronutrient levels. For example, a study on the impact of elevated CO_2_ on nine diverse rice cultivars showed that growth at elevated CO_2_ decreased the manganese (Mn) content in the body of rice plants by 53% (Ujiie et al., 2019). In this same study, the Mn content in the brown rice decreased by 7%, while the polished rice showed a 20.5% decrease in Mn when grown under elevated CO_2_ (Ujiie et al., 2019). The vast differences observed in Mn content in different tissues is a significant finding as rice is becoming an important forage crop in some regions of the world (Cheng et al., 2018). While Mn deficiency in forage animals is considered rare, such a significant decrease in micronutrients in the body of the plant suggests that forage animal nutrition will also suffer as a result of rising CO_2_. Thus, to accurately assess all the potential impacts of CO_2_-induced nutrient depletion on human health, it is crucial to measure nutrients in multiple plant tissues.

Interestingly, a more extensive meta-analysis of 130 plant species/cultivars was unable to detect a significant decrease in Mn content among C_3_ crops (Loladze, 2014). However, this study did identify significant decreases in many other micronutrients, namely iron (Fe) and zinc (Zn). Iron is of particular interest as at least 2 billion people currently suffer from Fe deficiency, making anemia a leading cause of maternal mortality (Micronutrient_Initiative, 2009). Zinc deficiency is also widespread, with approximately 30% of the world population at risk. Zinc deficiency can cause compromised immune responses, stunting during childhood, and increased risk of child mortality (Micronutrient_Initiative, 2009; Livingstone, 2015). While crosstalk between Fe, Zn, P, and S signaling in plants is recognized, not much is known in C_3_ or C_4_ plants regarding the mechanistic integration of these signaling networks (Mendoza-Cozatl et al., 2019; Xie et al., 2019). However, it was recently proposed that Fe, Zn, P, and S signaling are integrated in a PHR1 dependent manner in the C_3_ plant Arabidopsis (Briat et al., 2015). Interestingly, the rice homolog of PHR1, OsPHR2, was also shown to play a role in the integration of P and N signaling networks in rice (Hu et al., 2019). Thus, in C_3_ plants, it seems PHR proteins may be essential network hubs integrating signaling from multiple nutrients. When viewed from this perspective, the changes in micronutrient levels observed in C_3_ plants under elevated CO_2_ could be pleiotropic effects caused by disruption of N and/or P signaling. It remains unknown if these signaling networks are conserved between C_3_ and C_4_ plants.

Additionally, the genetic diversity in the C_3_ crops has a large impact on the effects of elevated CO_2_. The variation within species may even exceed the variation between species. For example, a study with 17 rice cultivars grown under controlled conditions in normal or 664 ppm CO_2_ showed 10–265% increase in total biomass and even greater—10–350% variation in response of grain yield (Ziska et al., 1996). This is true also for qualitative traits; protein content in grains of 18 field-grown rice cultivars cultivated at ca., 585 ppm CO_2_ decreased by 5–20%, whereas grain Zn and Fe concentrations decreased on average, but actually increased in four and two genotypes, respectively, and were not affected in another variety, Nipponbare (Zhu et al., 2018). Similar variation was observed in other species and, interestingly, modern varieties of oat, wheat, or soybean seem to be less responsive to elevated CO_2_ than varieties from the 1920s (Ziska and Blumenthal, 2007). There might, therefore, be a potential for the selection of new crop varieties for response to elevated CO_2_ levels (Shimono et al., 2018).

The question thus arises, can C_4_ crops help to alleviate “hidden hunger”? There are currently only five economically important C_4_ food crops—maize, sorghum, sugar cane, onion, and pearl millet. While the list of C_4_ crops is small, they account for a large proportion of global crop production. For example, the average annual production of maize from 2008–2010 was 750 million metric tons representing 27% of cereal area, 34% of cereal production and 8% of the value of all primary crop production (Shiferaw et al., 2011). The nutritional quality of these C_4_ crops is at best average, e.g., due to low lysine content in maize proteins or poor digestibility of sorghum and millet proteins (Millward, 1999; Galili and Amir, 2013). However, there are also several regionally important C_4_ crops, often called orphan crops, that have more desirable nutritional traits for combating hidden hunger. Notable orphan crops include grain amaranth, teff (*Eragrostis tef*), foxtail millet (*Setaria italica*), finger millet (*Eleusine coracana*), and proso millet (*Panicum miliaceum*).

These data suggest that while C_4_ crops do not show a CO_2_-induced nutritional penalty, the current staple C_4_ crops may not be best suited to address dietary deficits and hidden hunger. However, significant genetic advances have been made to improve the nutritional quality of maize and sorghum. For example, a recent genome-wide association study on 923 maize lines identified 46 QTLs significantly associated with seed Zn and Fe concentrations (Hindu et al., 2018). Introgressing favorable alleles of these QTLs into commercial varieties could improve both Zn and Fe levels in maize kernels. Additionally, researchers have developed quality protein maize (QPM), having almost twice the amount of lysine and tryptophan as traditional varieties, and maize lines with enhanced levels of provitamin-A or methionine (Wurtzel et al., 2012; Galili and Amir, 2013; Planta et al., 2017). Thus, biofortification is a viable approach to enhance the nutritional value of C_4_ crops and address hidden hunger.

In addition to food crops, there are eight C_4_ crops grown for turf, forage, or bioenergy. These include *Miscanthus* x *giganteus*, *Panicum virgatum* (switchgrass), *Chloris gayana* (Rhodes grass), *Cynodon dactylon* (Bermuda grass), *Melinis minutifolia* (molasses grass), *Panicum maximum*, *Cenchrus purpureus* (Napier grass), and *Zoysia japonica*. Collectively, these crops are all known for their high productivity and demonstrate the potential of C_4_ plants. Similar to food crops, a nutritional comparison of C_3_ and C_4_ forage grasses grown under high and low CO_2_ levels found that the C_3_ grasses had higher levels of protein, nonstructural carbohydrates, and water, but lower levels of fiber when grown under elevated CO_2_ compared to the C_4_ species (Barbehenn et al., 2004).

Under current environmental conditions, the staple C_4_ crops show superior productivity compared to C_3_ crops, and some of the C_4_ orphan crops seem to have the same or even better nutritional quality (**Table 1**). While the productivity gap can be expected to narrow down, due to elevated atmospheric CO_2_ that fertilizes C_3_ crops but not C_4_ crops, the relative nutritional value of the current C_4_ crops may improve because of the lack of the carbon nutrient penalty. Also the rise in temperatures may favor C_4_ crops in the future, or at least extend their cultivation areas. However, hidden hunger cannot be combatted without investment into further crop improvement, specifically targeting nutritional quality of staple C_4_ crops and improving the productivity of selected local crops with high nutritional value, such as pearl millet.

**Table 1:** Comparison of nutritional composition of grains of several cereal and orphan crops.

per 100 g DW	Crop	Energy (kcal)	Carbohydrate (g)	Protein (g)	Fat (g)	Ash (g)	Fiber (g)	Ca (mg)	Fe (mg)	Thiamin (mg)	Riboflavin (mg)	Niacin (mg)
C_3_	Rice (brown)	362	76	7.9	2.7	1.3	1	33	1.8	0.41	0.04	4.3
	Wheat	348	71	11.6	2	1.6	2	30	3.5	0.41	0.1	5.1
C_4_	Maize	358	73	9.2	4.6	1.2	2.8	26	2.7	0.38	0.2	3.6
	Sorghum	329	70.7	10.4	3.1	1.6	2	25	5.4	0.38	0.15	4.3
	Pearl millet	363	67	11.8	4.8	2.2	2.3	42	11	0.38	0.21	2.8
	Finger millet	336	72.6	7.7	1.5	2.6	3.6	350	3.9	0.42	0.19	1.1
	Foxtail millet	351	63.2	11.2	4	3.3	6.7	31	2.8	0.59	0.11	3.2
	Common millet	364	63.8	12.5	3.5	3.1	5.2	8	2.9	0.41	0.28	4.5
	Little millet	329	60.9	9.7	5.2	5.4	7.6	17	9.3	0.3	0.09	3.2
	Barnyard millet	300	55	11	3.9	4.5	13.6	22	18.6	0.33	0.1	4.2
	Kodo millet	353	66.6	9.8	3.6	3.3	5.2	35	1.7	0.15	0.09	2
	Teff	357	73	8–11	2.5	2.8	3	17 – 178	9.5 – 37.7	0.19	0.17	1.5
	Quinoa	399	67.6	12.9	5.8	2.2	13.6	148.7	13.2	0.13	0.02	0.6
	Grain Amaranth	371	65.3	13.6	7	2.9	6.7	159	7.6	0.116	0.2	0.92

Data are shown per 100 g dry weight and are taken from Caselato-Sousa and Amaya-Farfán (2012); Saleh et al. (2013), and Niro et al. (2019).

## Future Directions

### Open Questions on C_4_ Mineral Nutrition

**Figure 2:**
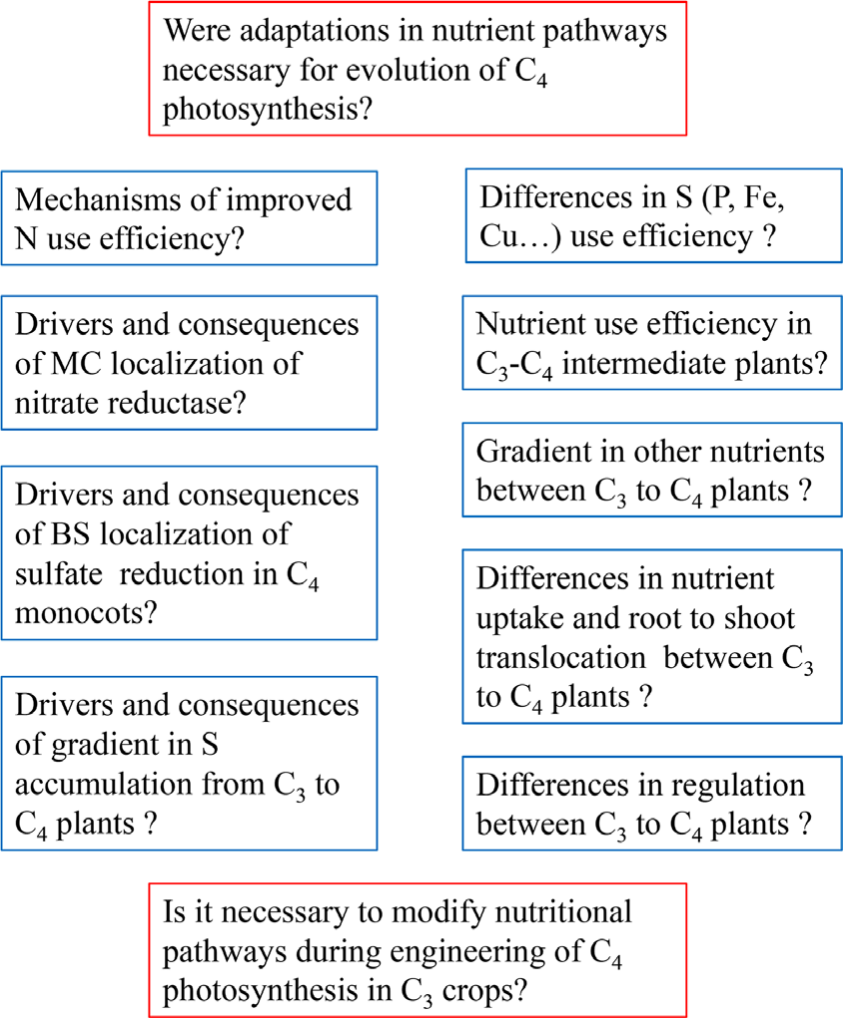
Summary of major research questions in mineral nutrition of C_4_ plants.

To improve the nutritional value of C_4_ crops for human food, it is necessary to understand more about the control of their nutrient homeostasis. While some progress has been made, e.g., in the biofortification of maize (Wurtzel et al., 2012; Galili and Amir, 2013; Planta et al., 2017), many questions on mineral nutrition of C_4_ plants are still open (**Figure 2**). Probably the biggest set of fundamental questions concerns the drivers and the consequences of the spatial separation of nitrate and sulfate assimilation in C_4_ monocots. Does the MC localization of nitrate reductase contribute to the improved nitrogen use efficiency of C_4_ plants? Have C_3_–C_4_ intermediate plants improved nitrogen use efficiency compared to C_3_ plants? Are there any differences in sulfur use efficiency between C_3_ and C_4_ plants? Is the gradient in the accumulation of sulfur compounds found in *Flaveria* conserved in other genera with C_3_ and C_4_ photosynthesis? Are the pathways of nitrate and sulfate assimilation differently regulated in C_3_ and C_4_ plants? Why is sulfate assimilation differently localized in C_4_ monocots and C_4_ dicots?

The other set of questions concerns other nutrients. Is there a gradient similar to that of sulfur compounds in *Flaveria* in accumulation of other nutrients between closely related C_3_ and C_4_ plants? Does the high flux through TPT and PPT in C_4_ plants affect their phosphate needs and homeostasis? Is phosphate homeostasis affected by elevated CO_2_? Is there a different need for Fe or Cu in C_4_ and C_3_ plants given the different arrangements of photosynthetic apparatus?

All these fundamental unknowns lead to one overarching question: Were adaptations in nutrient pathways necessary for the evolution of C_4_ photosynthesis? This question has major practical implications for the efforts to improve C_4_ crops, but particularly for engineering C_4_ photosynthesis to C_3_ crops.

### Improvement of C_4_ Crops by Traditional Breeding

Breeding material with high nutritional value is available for maize (Newell et al., 2014; Wang et al., 2019) and thus breeding for improved nutritional value is feasible. A few approaches for maintaining the nutritional levels of crops under elevated CO_2_ have been proposed. For example, the negative effect of elevated CO_2_ on nitrate assimilation and nitrogen content might be attenuated by increasing the proportion of ammonium as the nitrogen source (Bloom et al., 2010). However, crop species differ in their tolerance to ammonium, therefore, as discussed above, the most straightforward approach is to incorporate FACE studies into modern breeding programs. This approach would be useful for both C_3_ and C_4_ crops and would allow us to accomplish two goals. First, we could screen specifically for traits that improve the nutritional levels of crops under elevated CO_2_ and select for these traits in future cultivars. Secondly, we could ensure that traits selected to meet other breeding goals (i.e., pathogen resistance traits or drought resistance traits) are not negatively affected by elevated CO_2_ levels and do not further decrease the nutritional standards of our crops. While this approach would be technically challenging for breeding programs due to the expense and large space requirements associated with field-scale FACE studies, it has a high likelihood of success in the short term. As noted by Ujiie et al. (2019), carbohydrates, nitrogen, and sulfur resources are all transported through the phloem during nutrient reallocation and grain filling. Thus, improving nutrient translocation or the strength of the sink organ could counteract the nutritional decrease in crops grown under elevated CO_2_. These goals are well within the scope of modern breeding programs.

### C_4_ Engineering

The conversion of C_3_ crops to full C_4_ photosynthesis is a long-standing goal of plant biologists, and significant advances have been made with the help of both systems biology and synthetic biology (Schuler et al., 2016; Ermakova et al., 2020). To achieve this, at least five major milestones have been identified that are necessary to convert C_3_ crops to C_4_ photosynthesis: 1) induction of higher-order veins, 2) increase BS:M ratio, 3) adaptation of BS morphology, 4) engineering of dimorphic chloroplasts in BS and M cells, and 5) compartmentalization of the photosynthetic enzymes between BS and M cells (reviewed in Schuler et al., 2016). However, significant hurdles remain, especially in identifying a suitable C_3_ chassis for engineering, establishing Kranz anatomy, and the establishment of a carbon concentrating mechanism (Hennacy and Jonikas, 2020). Despite these challenges, consortiums like the C_4_ Rice Project, a global collaboration between leading researchers in photosynthesis, aim to engineer C_4_ photosynthesis into rice. Increasing rice yield and decreasing water and nitrogen fertilization requirements would significantly increase the sustainability of rice, a staple crop for 50% of the world population (see c4rice.com). Furthermore, additional C_3_ and C_4_ plant species are being developed for comparative studies to better understand the evolution of C_4_ traits. Potential model species of interest include the C_3_ panicoid grass *Dichanthelium oligosanthes*, which diverged from the C_4_ species *Setaria viridis* approximately 15 million years ago, representing a more recent divergence than most other C_3_ and C_4_ panicoid grasses (Studer et al., 2016).

An alternative to engineering C_4_ photosynthesis into C_3_ plants is using synthetic biology for improving photosynthesis (Kubis and Bar-Even, 2019). Possible mechanisms include engineering carbon concentrating mechanisms (Long et al., 2018), exploiting CAM mechanisms (DePaoli et al., 2014), or manipulating photorespiration (Maurino, 2019). Another possibility is to increase the performance of C_4_ crops directly. Indeed, it was possible to increase CO_2_ assimilation in maize by overexpressing Rubisco together with a chaperon, RUBISCO ASSEMBLY FACTOR 1 (RAF1), which resulted in fresh weight gain of the transgenic plants (Salesse-Smith et al., 2018). Alternatively, CO_2_ assimilation was increased by overexpression of Rieske FeS protein of the Cytochrome b6f complex in *Setaria viridis* (Ermakova et al., 2019). These efforts, however, concentrate fully on carbon fixation and do not consider the nutritional aspects, neither with respect to the crop nutritional value nor the mineral nutrient homeostasis and use efficiency of the new crops. Nevertheless, while engineering C_4_ crops is a very active area of research, it is unlikely to contribute significantly to food security or improved crop nutrition in the short term.

### C_2_ Engineering

Recently, Lundgren (2020) presented a compelling case for engineering C_2_ photosynthesis into C_3_ crop plants to improve photosynthetic performance in the face of climate change. The main argument made in favor of this approach is that C_2_ photosynthesis is a stable intermediate physiological state between C_3_ and C_4_ metabolism that increases net carbon assimilation under high temperatures (Monson, 1989; Bellasio and Farquhar, 2019). But, importantly, C_2_ photosynthesis does not require the complex anatomical changes associated with C_4_ photosynthesis (Lundgren, 2020). This strategy could be useful in improving crop yields (or in mitigating yield declines) in the medium term. However, it is unclear how C_2_ engineering will impact the nutritional status of crops, particularly under elevated CO_2_, and the photosynthetic nutrient use efficiency. To the best of our knowledge, there are no FACE experiments evaluating the effects of elevated CO_2_ on the yield or nutritional status of C_2_ plants. Despite these limitations, this approach seems feasible for two reasons. First, C_2_ engineering appears to be a necessary step toward C_4_ engineering, suggesting that these efforts will not be wasted in the long term. Secondly, even if initial C_2_ engineering has a negative impact on plant nutrition, when combined with traditional breeding approaches and additional engineering efforts, there is a high likelihood that these can be reverted. Thus, C_2_ engineering of C_3_ crops is likely to increase yield while maintaining or improving nutritional quality.

### *De Novo* Domestication

Of the approximately 150 commonly cultivated crops worldwide, humans obtain almost 50% of their calories from just three crops - rice, wheat, and maize (Ross-Ibarra et al., 2007). This is in stark contrast to preagricultural humans who had significantly more diverse diets and achieved some level of domestication in approximately 2,500 plant species (Khoury et al., 2014; Smykal et al., 2017). Recent advances in genome editing technology have made *de novo* domestication of wild plants a viable option to design ideal crops for the future (Fernie and Yan, 2019). For example, a recent study targeting a small number of critical genes in the orphan Solanaceae crop “groundcherry” (*Physalis pruinosa*) was able to rapidly improve plant architecture and productivity (Lemmon et al., 2018). Because groundcherry is a semi-domesticated orphan crop in the same family as tomato, researchers quickly identified homologues of two domestication genes—*SELF PRUNING 5* and *CLAVATA1.* Using genome editing techniques to induce mutations in these genes resulted in an increased fruit size of over 20% and improved plant architecture (more compact growth), making groundcherry easier to grow and harvest. Furthermore, advances in multiplexing platforms that allow simultaneous genome editing of six or more genes in a single transformation open the door for similar improvements to be made quickly in wild species (Zhang et al., 2016). Considering the small number of C_4_ plant species that have been domesticated and the growing list of known domestication genes to target, there is good reason to believe the weeds of today could be the nutritious and sustainable foods of tomorrow.

## Conclusions

C_4_ crops play an essential role in human nutrition, and this role will probably be even stronger in the future. They are characterized by high productivity and adaptability to warm and dry climates and by their better water and nitrogen use efficiency than C_3_ crops. While their yields will not directly benefit from elevated CO_2_, their nutritional value is not predicted to be negatively affected. However, to unlock the full potential of C_4_ crops for the future, more fundamental knowledge on the connection between mineral nutrition and C_4_ photosynthesis needs to be generated. As outlined above, in particular nitrogen metabolism underwent significant alterations in the course of evolution of C_4_ photosynthesis and might have been one of the evolutionary drivers. The increasing number and availability of new genomic and genetic resources and tools will enable us to extend the investigations of plant nutrition to a wider variety of C_4_ and C_3_–C_4_ intermediate species, and at the same time, to include investigations of nutrient homeostasis in the general framework of C_4_ photosynthesis research.

## Author Contributions

All authors contributed to the article and approved the submitted version.

## Funding

Research in SK’s lab is funded by the Deutsche Forschungsgemeinschaft (DFG) under Germany´s Excellence Strategy – EXC 2048/1 – project 390686111. IZ is supported by a DAAD fellowship.

## Conflict of Interest

The authors declare that the research was conducted in the absence of any commercial or financial relationships that could be construed as a potential conflict of interest.
